# Research Progress on Offline Defect-Detection Technologies for Selective Laser-Melting Parts

**DOI:** 10.3390/ma19204360

**Published:** 2026-10-16

**Authors:** Chenchen Tian, Kai Xu, Zikui Wu, Xuekun Li

**Affiliations:** 1 School of Mechanical Engineering, Shandong University, Jinan 250061, China; kaixu@mail.sdu.edu.cn (K.X.); zikuiwu@mail.sdu.edu.cn (Z.W.); 2 Key Laboratory of High Efficiency and Clean Mechanical Manufacture of Ministry of Education, Shandong University, Jinan 250061, China; 3 National Key Laboratory of High-End Equipment and Advanced Technology for Metal Forming, Shandong University, Jinan 250061, China; 4 Department of Mechanical Engineering, Tsinghua University, Beijing 100084, China

**Keywords:** selective laser melting, ultrasonic testing, XCT, defect detection

## Abstract

Selective laser melting (SLM) is recognized as one of the most promising metal additive manufacturing technologies for industrial deployment and has been applied to fabricate precision components for high-end equipment in sectors including aerospace and biomedicine. Nevertheless, ensuring reliable component quality and consistent performance remain a core challenge for SLM, which is widely acknowledged as a major barrier restricting the large-scale industrial application of the technology. Multi-scale and multi-type defects, which are difficult to fully suppress during the forming process, are the primary contributing factors to this issue. Accordingly, research on offline defect-detection technologies for SLM components is a critical pathway to break through the quality bottleneck and has emerged as a prominent research focus in both academia and industry. Based on a systematic review of relevant literature published in the past five years, this paper summarizes the morphological characteristics and formation mechanisms of typical defects in SLM-fabricated parts, including porosity, cracks, inclusions, and surface-forming defects. It elaborates on mainstream offline detection methods such as ultrasonic testing, X-ray computed tomography (XCT), and deep learning-enabled intelligent detection, and clarifies the respective performance advantages and inherent limitations of each technology. A quantitative comparison of these technologies is conducted from three core dimensions, detection accuracy, detection efficiency, and deployment cost, and differentiated application scenarios are defined corresponding to defect characteristics. Finally, the paper concludes with a full summary and prospects future in-depth research directions and industrial development trends in the field of SLM offline defect detection.

## 1. Introduction

Metal additive manufacturing (AM) is an emerging advanced manufacturing technology [1]. Using powder and wire as feedstocks and adopting electric arcs, electron beams, and lasers as heat sources, it realizes the near-net-shape fabrication of complex structures through a bottom-up layer-by-layer melting and deposition process [2]. This technology features remarkable advantages including wide material applicability, short production cycles, environmental friendliness, concentrated heat source energy, and exceptional suitability for processing components with complex geometries [3,4]. Recent studies further confirm that AM enables functionally graded and topology-optimized designs that are unattainable by conventional subtractive manufacturing, particularly for lightweight load-bearing structures [4,5,6].

According to their forming principles, metal AM technologies are mainly divided into two categories: powder bed fusion (PBF) and Directed Energy Deposition (DED) [7,8,9]. PBF refers to a technology that scans pre-deposited metal powder with a high-energy laser or electron beam along a predefined scanning path to fully melt the powder, followed by cooling and solidification to form components. Typical representatives include selective laser melting and Electron Beam Melting (EBM) [10]. In contrast, DED uses metal powder or wire as a raw material and high-energy lasers, electron beams, or plasma arcs as heat sources. The synchronously fed feedstock is rapidly melted and solidified along a predetermined processing path to achieve direct component fabrication, with Wire Arc Additive Manufacturing (WAAM) as a typical example [11].

As one of the metal AM technologies with the highest forming accuracy and most mature industrial applications, SLM has shown significant application value in high-end equipment fields such as aerospace and biomedicine, leveraging its core strength of integrated fabrication of complex structures. Typical aerospace applications include turbine blades, fuel nozzles, and structural brackets with conformal cooling channels, while biomedical applications encompass patient-specific implants and porous scaffolds with controlled porosity [12,13]. Nevertheless, the non-equilibrium melting and solidification characteristics coupled with multi-physics fields during the SLM process inevitably induce multi-scale and multi-type defects in components, including porosity, cracks, inclusions, and abnormal surface morphology. These defects severely restrict the mechanical property consistency and service reliability of as-built parts, and have become a core bottleneck limiting the large-scale industrial implementation of the technology [14].

Non-destructive testing (NDT) serves as a core approach for quality control and defect traceability of SLM components [15]. At present, a multi-modal technical system has been established, covering ultrasonic testing, XCT, and optical topography detection. The integration of deep learning has further promoted the intelligent upgrading of detection performance. It is important to clarify that these techniques are primarily employed in an offline/post-process manner, where inspection is performed after the build is completed and the part is removed from the powder bed. This differs fundamentally from in situ process monitoring, which uses layer-wise optical imaging or melt-pool signatures during deposition to infer probable defect formation in real time. Although in situ sensing delivers high temporal coverage, it usually yields only indirect evidence. By contrast, offline NDT provides direct post-build evidence and acts as the final quality gate. Representative in situ studies include machine learning classification of layer-wise images for flaw indication [16,17], which readers may consult for contrast. However, existing studies mostly focus on algorithm optimization and performance improvement of individual technologies, lacking systematic adaptability analysis tailored to different defect characteristics. In engineering practice, common issues include insufficient basis for technology selection and the difficulty of balancing the trade-off among accuracy, efficiency, and cost [18].

Therefore, to achieve efficient and precise quality control of SLM components, efforts should not be limited to optimizing the performance of a single detection technology. It is more critical to establish the mapping between defect characteristics and detection technologies and develop actionable guidelines for technology selection. Against this background, this paper reviews the relevant literature on defect detection for SLM-fabricated metallic parts published in the past five years based on previous research findings. It conducts a quantitative comparison of various detection technologies from three core dimensions, detection accuracy, efficiency, and deployment cost, and defines optimal application scenarios for each technology corresponding to different defect characteristics. This work systematically summarizes the research progress and application status in this field, aiming to provide references for scholars engaged in in-depth research on SLM defect-detection technologies.

### Literature Search and Review Methodology

Three major bibliographic databases were adopted for literature retrieval: Web of Science Core Collection, Scopus, and ScienceDirect. In addition, CNKI was searched for relevant Chinese-language publications. The time frame was constrained from January 2014 to July 2026. Search terms combined synonyms for manufacturing technology and defect-detection techniques. Topic search was performed on title–abstract–keywords: selective laser melting, laser powder bed fusion, SLM, defect detection, non-destructive testing, X-ray computed tomography, ultrasonic testing, deep learning, offline inspection.

Inclusion criteria:

1. Peer-reviewed original research articles or review papers related to offline non-destructive defect detection for SLM/L-PBF metallic components.
2. Studies reporting experimental data, detection performance, comparative analysis of ultrasonic testing, XCT, optical inspection, or deep learning-based inspection.
3. Publications containing measurable information on defect morphology, detection accuracy, inspection efficiency, or application-related cost.

Exclusion criteria:

1. Research only focusing on in situ monitoring/online sensing without offline defect-detection content.
2. Papers only discussing process parameter optimization or microstructure modification, lacking defect-detection evaluation.
3. Conference abstracts, patents, and preprints without formal peer review.
4. Non-English papers without accessible full-text English abstract and experimental descriptions.

All tables and figures in this manuscript were synthesized, summarized, or redrawn based on data extracted from the above-included literature. Schematic diagrams showing detection principles are original illustrations drawn by the authors. Defect micrographs were adapted from open access CC-BY-4.0 MDPI references, with full citation information provided in each figure caption.

The search was restricted to peer-reviewed journal articles; reviews were also eligible. Conference proceedings and patents were not prioritized in the primary search. The initial database search yielded 1287 records. After duplicate removal, 975 unique records remained. In the first-stage screening based on titles and abstracts, irrelevant documents were eliminated, including conference abstracts, patents, book chapters, studies focusing merely on in situ monitoring, pure process-optimization papers without defect-detection content, and simulation works without experimental validation. This screening step retained 247 candidate papers. Subsequently, full-text evaluation was conducted on these 247 papers. After applying predefined inclusion and exclusion criteria, 93 papers were finally incorporated for qualitative analysis and comparison in this review.

## 2. Typical Defects and Formation Mechanisms of SLM Components

Defects represent one of the most critical bottlenecks restricting the widespread industrial application of SLM technology. To overcome this bottleneck, it is essential to gain an in-depth understanding of the physical processes involved in SLM and conduct systematic research on defect types and their generation pathways. A clear clarification of defect categories and formation mechanisms can provide theoretical fundamentals for defect suppression and quality regulation. Typical defects in SLM-fabricated components include porosity, cracks, surface-forming defects, inclusions, and delamination. Table 1 summarizes the typical defects of SLM parts and their corresponding formation mechanisms reported in existing literature. The subsequent sections elaborate on various defects and their formation processes in detail and conclude the influences of key process parameters on each type of defect.

### 2.1. Morphological Characteristics and Formation Mechanisms of Porosity Defects

Porosity is the most prevalent and fundamental type of internal defect in SLM-fabricated components, which mainly manifests as cavities and void structural flaws within the material matrix [34]. Compared with conventional manufacturing processes such as casting and forging, the layer-by-layer melting and rapid solidification characteristics of SLM readily lead to issues including melt-pool instability, insufficient melt filling, and gas entrapment, which eventually induce porosity defects with varying morphologies, sizes, and distribution features. Such defects are also one of the primary causes of scattered mechanical properties and degraded fatigue reliability in additively manufactured parts.

**Figure 1:**
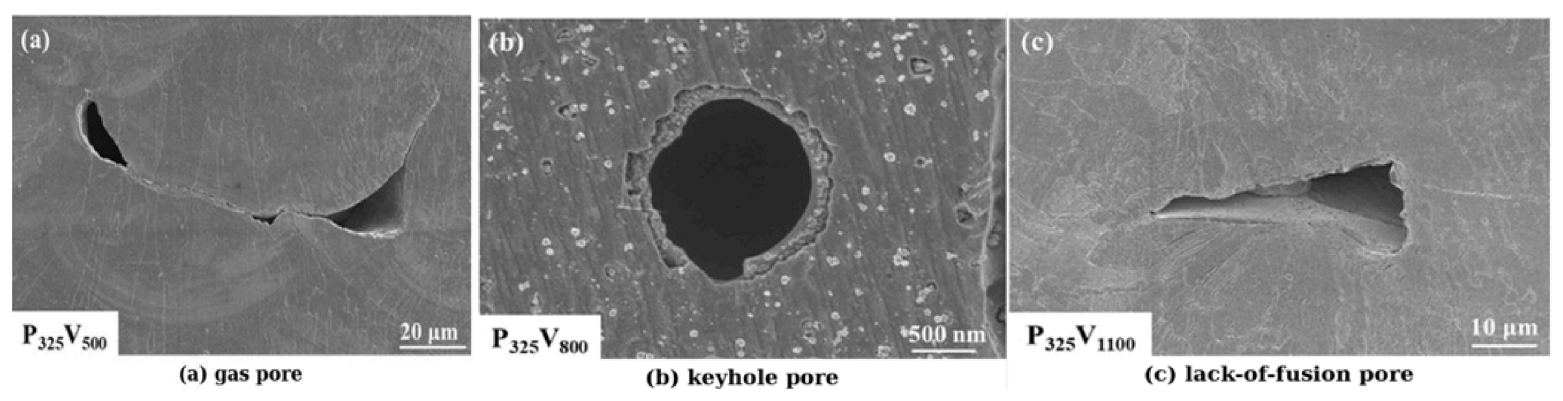
Typical pore defects in SLM parts: (**a**) gas pore; (**b**) keyhole pore; (**c**) lack-of-fusion pore. Micrographs are adapted from Ref. [27], licensed under a Creative Commons Attribution (CC-BY-4.0) license.

Based on differences in morphological characteristics and formation mechanisms, porosity formed by SLM can be mainly classified into three categories: gas porosity, lack-of-fusion porosity, and keyhole porosity. The micromorphologies and generation mechanisms of these three defect types are notably distinct, as illustrated in Figure 1.

Gas porosity accounts for the largest proportion of porosity defects in SLM-fabricated components. Its micromorphology typically presents as regular spherical or near-spherical shapes, with pore sizes concentrated in the range of several to tens of micrometers. The pore walls are smooth without distinct edges, and the pores exhibit a discrete, random distribution within the as-built matrix. In essence, this type of defect forms as closed cavities when gaseous phases in the melt pool are trapped and unable to escape due to rapid solidification [28]. The gas sources can be summarized into three categories: moisture adsorbed during powder storage and residual gas from the atomization process, bubbles entrained into the melt pool by the inert shielding atmosphere in the building chamber, and metal vapor generated by the vaporization and oxidation of alloying elements in the high-temperature melt pool. AN et al. [29] systematically summarized the complete formation and evolution mechanism of spherical gas pores under the SLM process. They pointed out that the extremely high non-equilibrium solidification rate of SLM greatly narrows the window for bubble buoyancy and escape. Since the propagation rate of the melt-pool solidification front is significantly higher than the bubble migration rate, bubbles formed by various gaseous components are encapsulated by molten metal and permanently trapped inside the matrix, eventually forming spherical gas pore defects. Although individual micropores are small in volume and highly dispersed in space and generally do not directly cause instantaneous fracture failure of components, they disrupt the continuity of the metal matrix and induce local stress concentration under cyclic loading, acting as preferential initiation sites for fatigue cracks.

Lack-of-fusion (LOF) porosity mostly appears as irregular elongated or slit-like shapes with rough edges and sharp corners [30,31]. It features a wide size range and is spatially concentrated at interlayer bonding interfaces and melt track overlap zones, posing significantly more harm than spherical gas pores. In terms of formation mechanism, such defects arise from insufficient laser energy input or a low melt track overlap rate, which result in incomplete powder melting and failure to form effective metallurgical bonding between adjacent melt pools [32]. Mesoscopic numerical simulations can fully reproduce its initiation process. Bauereiß et al. [22] established a lattice Boltzmann model coupled with the thermocapillary effect and solid–liquid wetting behavior. The model reproduced the evolution of restricted melt spreading under low energy input at the powder particle scale, clearly revealed the formation and growth rules of lack-of-fusion voids at interlayer and melt track overlap regions, and hydrodynamically confirmed that insufficient wetting is the core internal cause inducing irregular slit-like porosity. Experimental conclusions are highly consistent with simulation results. Through controlled process parameter experiments, Trithepchunlayakoo [19] found that an excessively high laser scanning speed, low laser power, or excessive powder layer thickness reduces both the width and depth of a single melt track. Unmelted powder regions remain between adjacent melt tracks and across building layers, and the melt cannot fully fill the gaps, forming slit-like lack-of-fusion porosity after cooling and solidification. In addition, issues such as uneven powder distribution and powder agglomeration during the spreading process further increase the occurrence probability of such defects [35]. Due to their sharp morphology and weak interfacial bonding, lack-of-fusion pores are typical crack-like defects with much stronger stress concentration effects than regular gas pores. Under static and cyclic fatigue loading, they easily serve as initiation sites for crack propagation, directly impairing the load-bearing capacity and structural integrity of components.

Keyhole porosity is a typical defect in the high-energy-density processing window of SLM [33,37]. It mostly manifests as large-sized irregular deep holes or even through-holes, with complex morphology and highly dispersed pore sizes. The pore walls are often accompanied by local collapse and uneven features [38]. Regarding its formation mechanism, WU et al. [23] concluded through recoil pressure-coupled simulation analysis that this defect is directly related to keyhole instability in the laser deep-penetration mode. When the laser power density is too high and the scanning speed is too slow, the metal material violently vaporizes instantaneously, and the generated high-intensity vapor recoil pressure continuously squeezes the melt pool, forming a deep keyhole structure. Once process parameter matching is out of balance and melt-pool fluid stability is disrupted, the keyhole will close and collapse. The melt cannot backfill the cavity in time, and eventually large-sized keyhole pores are trapped inside the component. Compared with gas porosity and lack-of-fusion porosity, keyhole porosity has a larger overall scale and more concentrated spatial distribution. It directly reduces the effective load-bearing cross-sectional area of the component and induces strong stress concentration at defect edges, making it a high-risk defect that causes brittle fracture and premature fatigue failure of SLM parts [38].

Overall, the morphological regularity, size, and spatial distribution of SLM porosity defects are entirely determined by laser energy input, melt-pool dynamic behavior, and process parameter matching. The hazard degree of different types of porosity varies significantly. Accurate differentiation of the morphological characteristics and formation mechanisms of various porosity types serves as an important theoretical basis for targeted defect detection, process optimization, and component quality evaluation.

### 2.2. Characteristics and Metallurgical Mechanisms of Crack and Delamination Defects

Cracks and delamination are highly detrimental structural defects formed during the SLM process. Distinct from porosity-type void defects, they typically exhibit penetrating and interfacial debonding damage characteristics, directly disrupting the matrix continuity and interlayer bonding strength of components. They serve as the primary cause of catastrophic failure of SLM precision parts under load-bearing and cyclic service conditions. The extremely high heating and cooling rates of the SLM process induce severe temperature gradients and cyclic thermal stress inside components. Coupled with the layer-by-layer deposition feature, this readily triggers issues such as intergranular cracking and interlayer delamination. Based on defect morphology, distribution location, and formation mechanism, such defects can be classified into two typical categories: microcracks and interlayer delamination [23].

**Figure 2:**
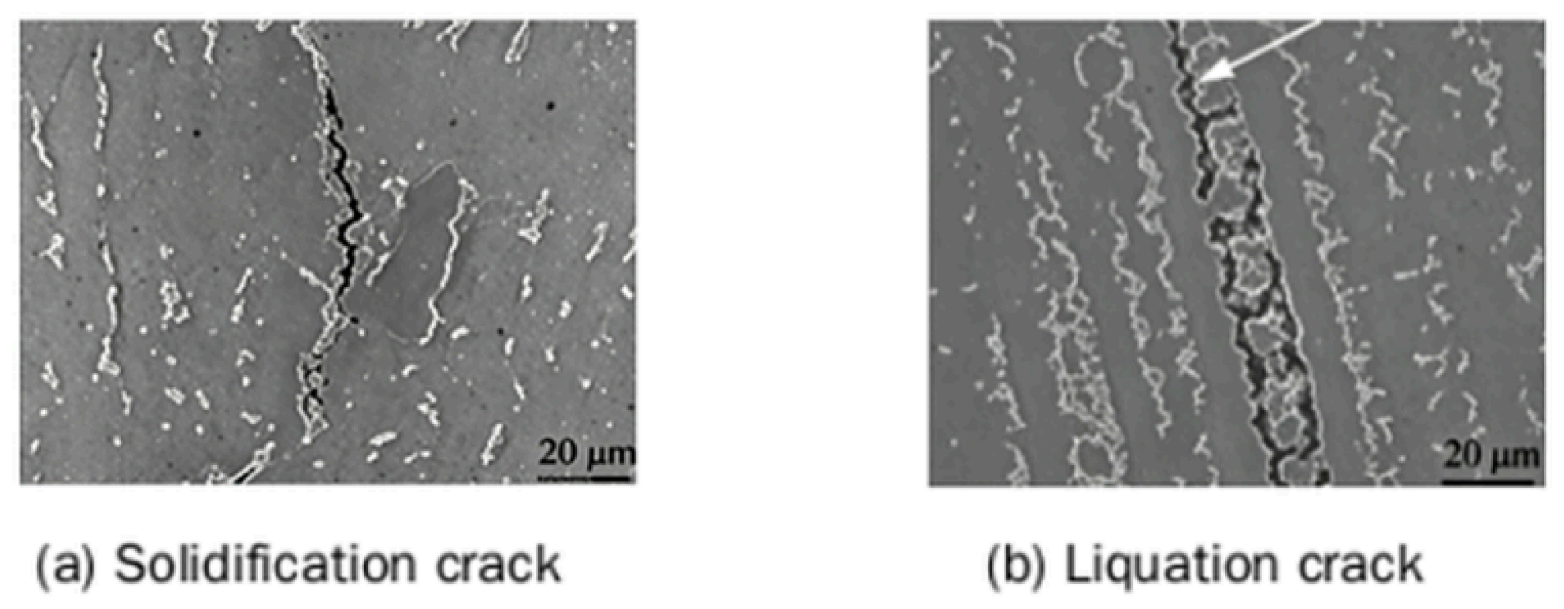
Typical cracks in SLM-fabricated components: (**a**) solidification crack; (**b**) liquation crack. White arrow denotes liquation crack. Micrographs are adapted from Ref. [12], published under a CC-BY-4.0 license.

Microcracks generated by SLM mainly consist of solidification cracks and liquation cracks [39,40]. They mostly exist as tiny microscopic gaps distributed along grain boundaries or interdendritic regions, with lengths ranging from micrometers to hundreds of micrometers, and feature high concealment and strong propagation tendency [37]. Typical solidification cracks and liquation cracks are shown in the Figure 2. Solidification cracks mainly form at the final stage of melt-pool solidification. During the rapid solidification of melt-pool metal, grains grow continuously and interconnect with each other, and the residual liquid phase is squeezed and isolated at grain boundary regions, forming continuous or discontinuous liquid films. Under the coupled action of solidification shrinkage stress and thermal stress, the grain boundary liquid films cannot effectively compensate for the shrinkage deformation of the matrix. Eventually, the grain boundaries are pulled apart, forming intergranular cracks [41]. Liquation cracks mostly occur in the partially melted zone surrounding the melt pool. In this region, low-melting-point alloy phases at grain boundaries re-liquefy under laser thermal action, forming local grain boundary liquid films. During the subsequent cooling and shrinkage process, the liquid films fail to maintain the grain boundary bonding strength, and microcracks initiate under thermal stress. Such cracks are particularly prevalent in crack-susceptible materials such as superalloys and high-strength steels [20].

It is widely acknowledged in existing studies that increasing laser energy density can effectively suppress porosity defects and reduce the void fraction in SLM-built components. Nevertheless, the evolution behavior of cracks differs remarkably from that of porosity defects and cannot be simply regulated by adjusting a single process parameter. Carter et al. [42] conducted full-factorial controlled experiments on two γ′-strengthened nickel-based superalloys, CM247LC and CMSX486. The results confirmed that although the porosity fraction of components continuously decreases with increasing laser energy density, there is no direct monotonic correlation between crack density and energy density. The study further indicated that the initiation of SLM hot cracks results from the coupling effect of metallurgical properties and mechanical behavior. Intrinsic metallurgical characteristics of materials, such as the solidification temperature range, together with mechanical factors including residual stress, collectively determine cracking susceptibility. Guo et al. [43] further found that residual stress and the solidification time of molten metal are the dominant factors affecting crack density. Specifically, crack density decreases with increasing hatch spacing and scanning speed and increases with rising laser power.

**Figure 3:**
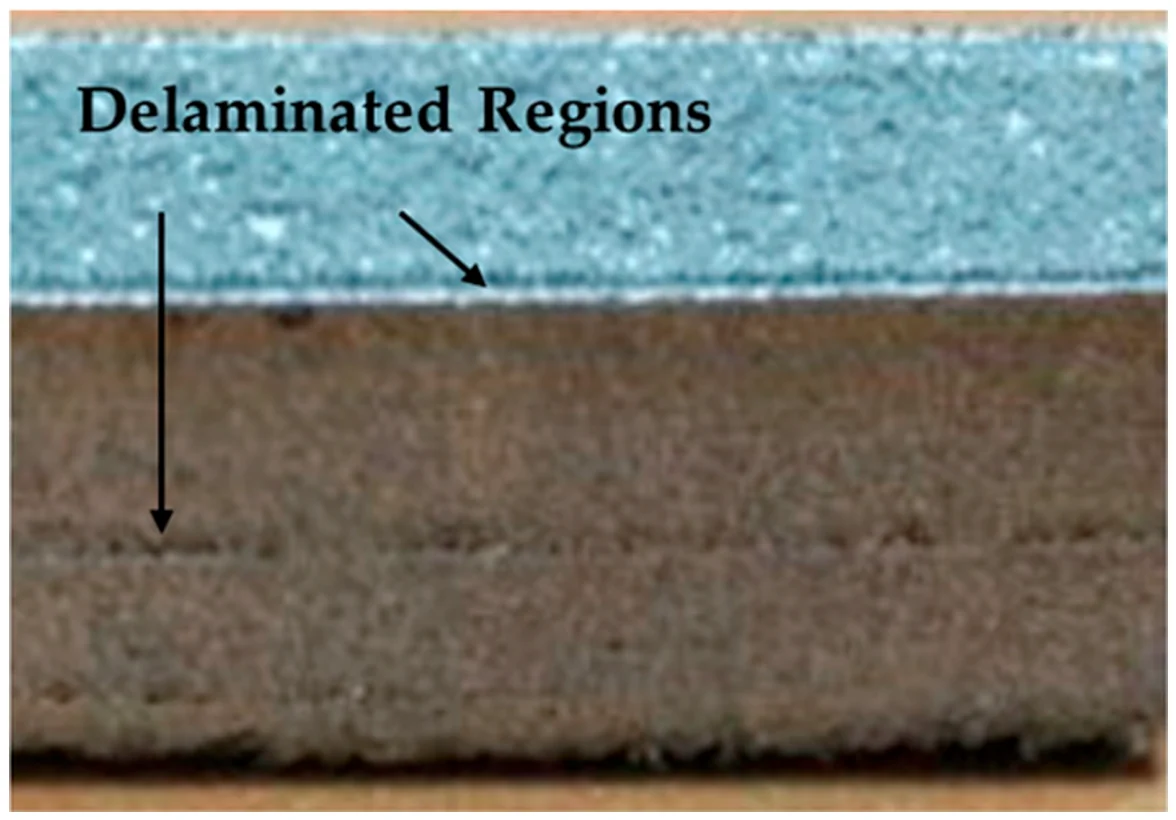
Optical micrograph showing the delaminated regions in the SLM sample. The arrows indicate the locations of delamination defects. (micrograph adapted from Ref. [12], licensed under CC-BY-4.0 open access license).

Delamination is a unique interfacial defect inherent to the layer-by-layer deposition process of SLM [21]. It is mainly manifested as local or overall separation and interfacial debonding between adjacent building layers. The defects are mostly distributed parallel to the building substrate, presenting sheet-like or band-like gap features with a width far larger than that of conventional microcracks. As shown in the Figure 3, such defects directly impair the interlayer mechanical bonding performance of components [25]. The core formation mechanism of delamination defects is insufficient interlayer metallurgical fusion. When the laser energy density is inadequate, the single-layer powder thickness is excessively large, or the matching of interlayer scanning paths is poor, the penetration depth of the upper melt pool cannot fully cover the underlying formed layer. As a result, an effective metallurgical bonding interface cannot be formed between the upper and lower layers, and the layers are only connected by the mechanical stacking of incompletely melted powder. Meanwhile, repeated rapid heating and cooling during the SLM process accumulate residual tensile stress inside the components. Since the interlayer interface is a mechanically weak region, the sustained action of residual stress will further pull apart the incompletely bonded interlayer interface and aggravate the expansion and propagation of delamination defects. In addition, surface flaws such as powder adhesion, protuberances, and micro-pits on the surface of previously formed layers will cause uneven powder spreading in subsequent layers, further hindering the effective fusion of interlayer melts and increasing the occurrence probability of delamination defects.

Compared with porosity defects, crack and delamination defects cause more prominent structural damage. Microcracks can propagate continuously and coalesce with each other even under extremely low cyclic loads, and gradually evolve into macroscopic through-thickness cracks. Delamination defects directly undermine the interlayer integrity of components, resulting in significantly aggravated mechanical property anisotropy and greatly reduced tensile, impact and fatigue performance, which severely restricts the engineering application of SLM components in load-bearing structures of high-end equipment. Clarifying the metallurgical formation mechanisms of these two types of defects is a critical prerequisite for realizing crack suppression, enhancing interlayer bonding, and improving the structural integrity of components.

### 2.3. Inclusions, Microstructural Abnormalities, and Surface-Forming Defects

**Figure 4:**
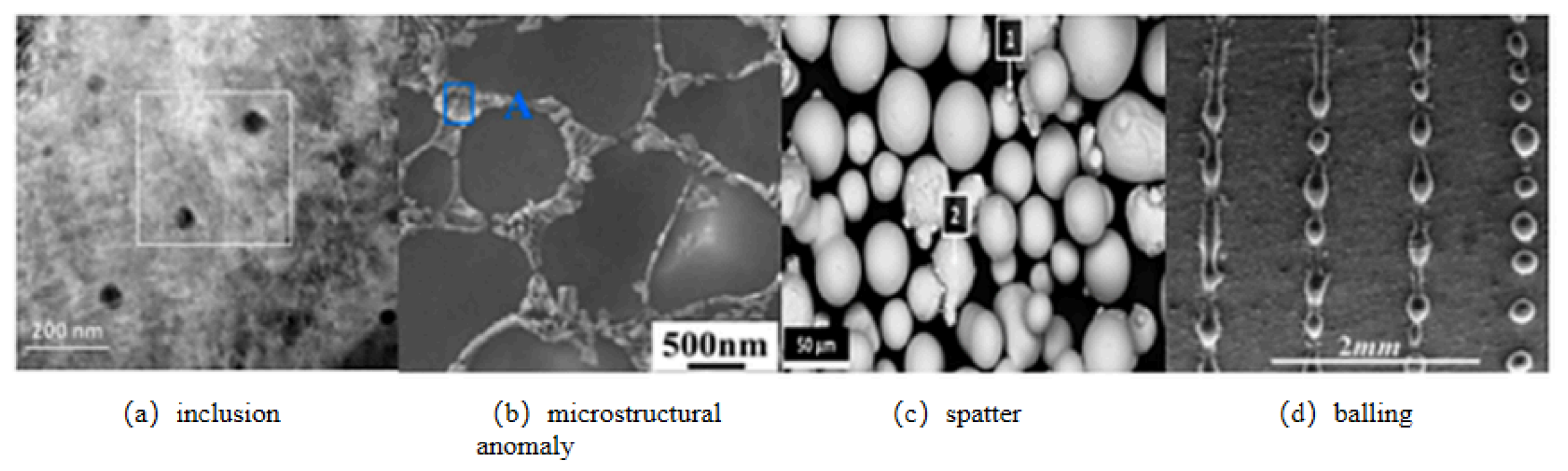
Typical defects in laser powder bed fusion (LPBF). (**a**) Inclusion: TEM image showing nanoscale inclusions dispersed in the matrix. (**b**) Microstructural anomaly: SEM image of the solidified cellular/grain structure; the blue circle highlights the anomalous region, and the letter A indicates its position. (**c**) Spatter: optical micrograph of spatter particles ejected from the melt pool and deposited on the powder bed; the white arrows point to typical spatter droplets/satellites. (**d**) Balling. (micrographs (**a**,**b**,**d**) are adapted from Ref. [45], and micrograph (**c**) is adapted from Ref. [46], all licensed under CC BY 4.0 open access licenses.).

In addition to typical structural defects such as porosity, cracks, and delamination, the SLM process is also prone to inclusion defects, microstructural abnormalities, and various types of surface-forming defects, as illustrated in Figure 4. Although such defects rarely cause instantaneous fracture failure of components, they persistently impair the microstructural homogeneity and surface integrity of the material, and degrade the wear resistance, corrosion resistance, and fatigue resistance of parts. They serve as important contributing factors to the poor service stability and large performance dispersion of SLM components, and together with internal void defects constitute a complete defect system [44].

Inclusion defects are typical metallurgical impurity-type defects in SLM fabrication, dominated by hard non-metallic inclusions such as oxides, nitrides, and carbides [47,48]. They mostly present punctate, flocculent, or irregular block-like morphologies and are dispersedly distributed within the matrix, with sizes ranging from submicron to tens of micrometers [49]. Their formation mainly stems from two sources: first, oxidation and moisture absorption of powder feedstock during storage and delivery, where oxide films formed on powder surfaces enter the melt pool during melting and remain in the matrix after solidification; second, insufficient shielding atmosphere purity during the building process, which triggers metallurgical reactions between the high-temperature melt pool and residual oxygen/nitrogen, forming hard inclusion phases in situ. Unlike porosity void defects, inclusion defects possess certain structural strength, but they disrupt the continuity of the metal matrix and form stress concentration sites at the interface between the inclusion phase and the matrix. These sites readily induce the initiation and propagation of microcracks, while significantly reducing the plasticity and toughness of the material and aggravating the mechanical property anisotropy of components.

Microstructural abnormality is a characteristic defect caused by the rapid non-equilibrium solidification of SLM, mainly manifested as grain coarsening, disordered grain orientation, precipitate phase segregation, and inhomogeneous grain boundary structure [23]. The temperature gradient effect induced by localized laser heating and ultra-fast cooling drives grains inside the melt pool to grow preferentially along the heat conduction direction, forming a coarse columnar grain structure and breaking the uniformity of grain size and orientation. Meanwhile, alloying elements have insufficient time for full diffusion during rapid solidification, which easily leads to the enrichment of low-melting-point elements and hard precipitates at grain boundaries, causing composition segregation and microstructural inhomogeneity. For instance, research by Bitharas [24] shows that during the high-energy-density processing of Ti-6Al-4V(TC4) alloy, aluminum element undergoes significant vaporization loss with a decreased content. Such composition deviation directly alters the solidification temperature range and phase transformation behavior of the alloy, further exacerbating composition segregation and microstructural inhomogeneity. Although these microstructural abnormalities cause no obvious macroscopic structural damage, they directly change the intrinsic mechanical and physical properties of the material, resulting in the uneven distribution of hardness, strength, and wear resistance, and greatly reducing the service performance stability and reliability of components.

Surface-forming defects are the most intuitive external defects of SLM components, covering various morphologies such as powder adhesion, metal spatter, melt track undulation, balling, and burrs, which directly determine the surface quality and subsequent machining allowance of parts. Among them, balling and spatter are the most common: imbalanced laser energy density and melt-pool fluid instability cause melt agglomeration that adheres to the surface, forming balling defects; metal vapor recoil in the melt pool and shielding gas flow disturbance lead to melt spatter, which further deteriorates surface flatness [36]. Issues such as uneven powder spreading, unreasonable scanning paths, and interlayer fusion differences will also aggravate defects including melt track undulation, local powder adhesion, and surface pores. Surface-forming defects directly increase the surface roughness of components and damage surface integrity. They not only raise the cost of subsequent post-processing procedures such as grinding and polishing, but also form stress concentration at surface defects, serving as initiation sources of fatigue cracks and significantly shortening the service life of parts. Research by Yanez et al. [26] on SLM-fabricated TC4 gyroid structures confirms that almost all fatigue cracks originate from surface defects, and the notch effect caused by surface defects is the main cause of fatigue failure, markedly reducing the service life of components. At present, surface quality can be effectively improved by adjusting process parameters such as laser power, scanning speed, and powder layer thickness. Although post-processing can further enhance surface precision, it increases manufacturing costs and reduces production efficiency. Therefore, more efficient, novel, in situ regulation methods for surface quality still need to be explored.

In summary, inclusion defects, microstructural abnormalities, and surface-forming defects fall into three categories, respectively: internal metallurgical impurities, microstructural distortion, and external morphological damage. Their formation mechanisms correspond to atmospheric metallurgical reactions, microstructural evolution during non-equilibrium solidification, and melt-pool dynamics plus powder spreading process stability. The three types of defects are interrelated and mutually influential: surface defects tend to induce internal microstructural damage, while microstructural abnormalities and internal inclusions further deteriorate the surface-forming quality, jointly restricting the forming quality and service performance of SLM components.

## 3. Research Progress of Non-Destructive Testing Technologies for SLM Defects

### 3.1. Ultrasonic Testing Technology for Internal Defects

**Figure 5:**
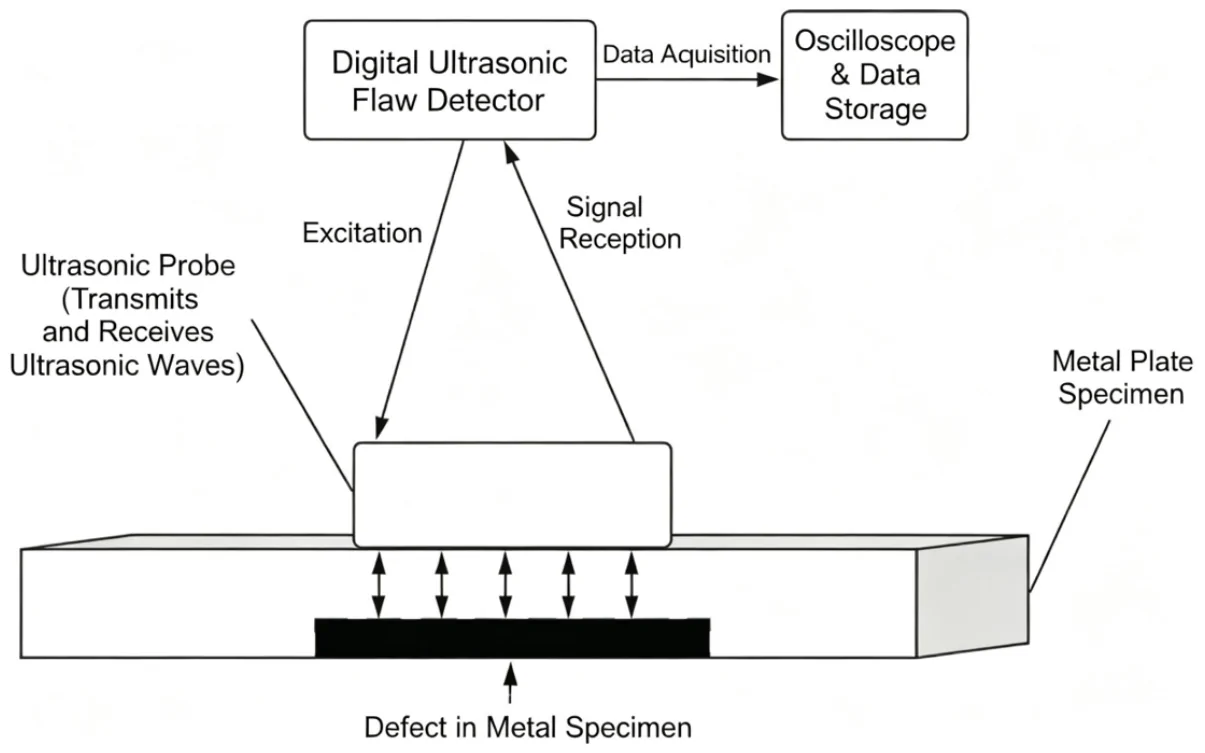
Schematic diagram of the ultrasonic testing system.

During SLM fabrication, defects of varying types, sizes, morphologies, and spatial locations generate acoustic signals with distinct characteristic signatures [50]. The selection of appropriate transducers for signal acquisition and the accurate classification of corresponding defect categories constitute the core challenge in SLM defect detection. To address the multi-scale, multi-morphology, and spatially complex nature of defects in SLM components—frequently featuring intricate curved surfaces and internal cavity structures—existing research has gradually established two complementary technical routes: conventional piezoelectric ultrasonics and laser ultrasonics. These approaches complement one another in detection accuracy, deployment cost, and applicable scenarios, jointly supporting the internal defect inspection requirements of SLM components. The working principle of the ultrasonic testing system is illustrated in Figure 5.

Conventional piezoelectric ultrasonic testing is a well-established technology with low equipment cost and high detection efficiency, and is currently the preferred solution for batch offline quality inspection of SLM components in industrial scenarios. Its fundamental working architecture relies on a piezoelectric transducer to generate ultrasonic waves, which are coupled into the interior of the component via a couplant. The echo signals reflected from defects are then collected and converted into electrical signals for subsequent analysis [51]. Given the coarse grain structure and strong acoustic attenuation of SLM-fabricated materials, existing studies mainly improve detection performance from two dimensions: transducer structure optimization and imaging method upgrading. In terms of transducer optimization, Shi et al. [52] adopted a 10 MHz water-immersion focused probe to inspect SLM titanium alloy specimens, achieving effective identification of internal gas pores and lack-of-fusion defects. Li et al. [53] further developed an annular array ultrasonic transducer, which delivers higher accuracy in defect morphology characterization than traditional linear array transducers, and is more compatible with C-scan imaging of high-attenuation additive manufactured materials. From the perspective of technical applicability, conventional ultrasonic testing is suitable for SLM components with relatively regular geometries. It enables rapid preliminary screening of internal defects in large-volume components and simultaneously supports semi-quantitative characterization of residual stress. Nevertheless, its inherent limitations are prominent. The contact-based detection mode depends on couplants, making it incompatible with components featuring complex curved surfaces, closed inner cavities, and miniature dimensions. A detection blind zone exists in the near-surface region, hindering the identification of subsurface defects. Restricted by transducer frequency, it has insufficient resolution for microporosity and microcracks smaller than 0.5 mm and thus cannot satisfy the high-precision detection requirements of high-end precision components.

Constrained by its contact detection mode and upper resolution limit, conventional piezoelectric ultrasound fails to meet the detection demands of components with complex geometries and tiny internal defects. With the merits of non-contact operation, high spatial resolution, and adaptability to high-temperature conditions, laser ultrasound has gradually emerged as a vital research direction in the field of SLM micro-defect detection. In the area of imaging algorithm optimization, the synthetic aperture focusing technique (SAFT) is the most widely adopted imaging enhancement method. Lévesque et al. [54] combined laser ultrasound with the synthetic aperture focusing algorithm and realized the identification of porosity and lack-of-fusion defects in Inconel 718 (IN718) and TC4 alloy specimens, with detection results in good agreement with micro-CT findings. This scheme verified the quantitative detection capability of laser ultrasound for typical SLM defects, but it was only validated on flat specimens of two alloys without considering the interference of surface roughness and residual stress on acoustic signal propagation. Its versatility in multi-material and complex structure scenarios remains inadequate. In the area of signal processing and feature extraction, since laser ultrasonic signals are vulnerable to environmental noise and surface diffuse reflection, efficient noise reduction and feature extraction algorithms are critical for improving detection reliability. Klein et al. [55] introduced a wavelet analysis method to perform time-domain separation of laser ultrasonic surface wave signals from a 316 stainless-steel and titanium alloy, which effectively enhanced defect features in B-scan images and improved defect positioning accuracy. This approach confirmed the performance improvement brought by intelligent signal processing algorithms for ultrasonic detection. In the area of system architecture and performance upgrading, to address the demands of SLM in situ detection and high-precision inspection, researchers have further developed high-temperature-compatible laser ultrasonic detection systems and high-resolution imaging schemes. For instance, Chen et al. [56] proposed a high-resolution imaging scheme based on pulsed laser excitation and optical detection, which can identify 0.5 mm-scale subsurface pores under high-temperature environments, breaking through the temperature limitation and near-surface blind zone of conventional ultrasound.

Overall, conventional ultrasound and laser ultrasound form a complementary technical landscape: the former is mature and cost-effective, applicable to offline batch preliminary screening; the latter features higher accuracy and non-contact operation, with greater potential for on-line monitoring and inspection of complex components. However, both technologies currently share common shortcomings. First, existing studies mostly focus on laboratory validation of regular flat specimens, while systematic research on typical complex curved surfaces and inner cavity structures of SLM components is insufficient. Second, the microstructural anisotropy and surface roughness of SLM materials cause significant interference to ultrasonic signal propagation, resulting in unstable accuracy of quantitative defect identification. Third, the high equipment cost and complex data processing procedures of laser ultrasound restrict its industrial implementation. Future research should further optimize adaptive signal noise reduction algorithms under complex working conditions, construct an acoustic feature database for defects across multiple materials, and promote the integration of ultrasonic testing and deep learning, so as to improve the accuracy and efficiency of intelligent defect identification.

### 3.2. XCT-Based High-Precision Testing Technology for Micro-Defects

**Figure 6:**
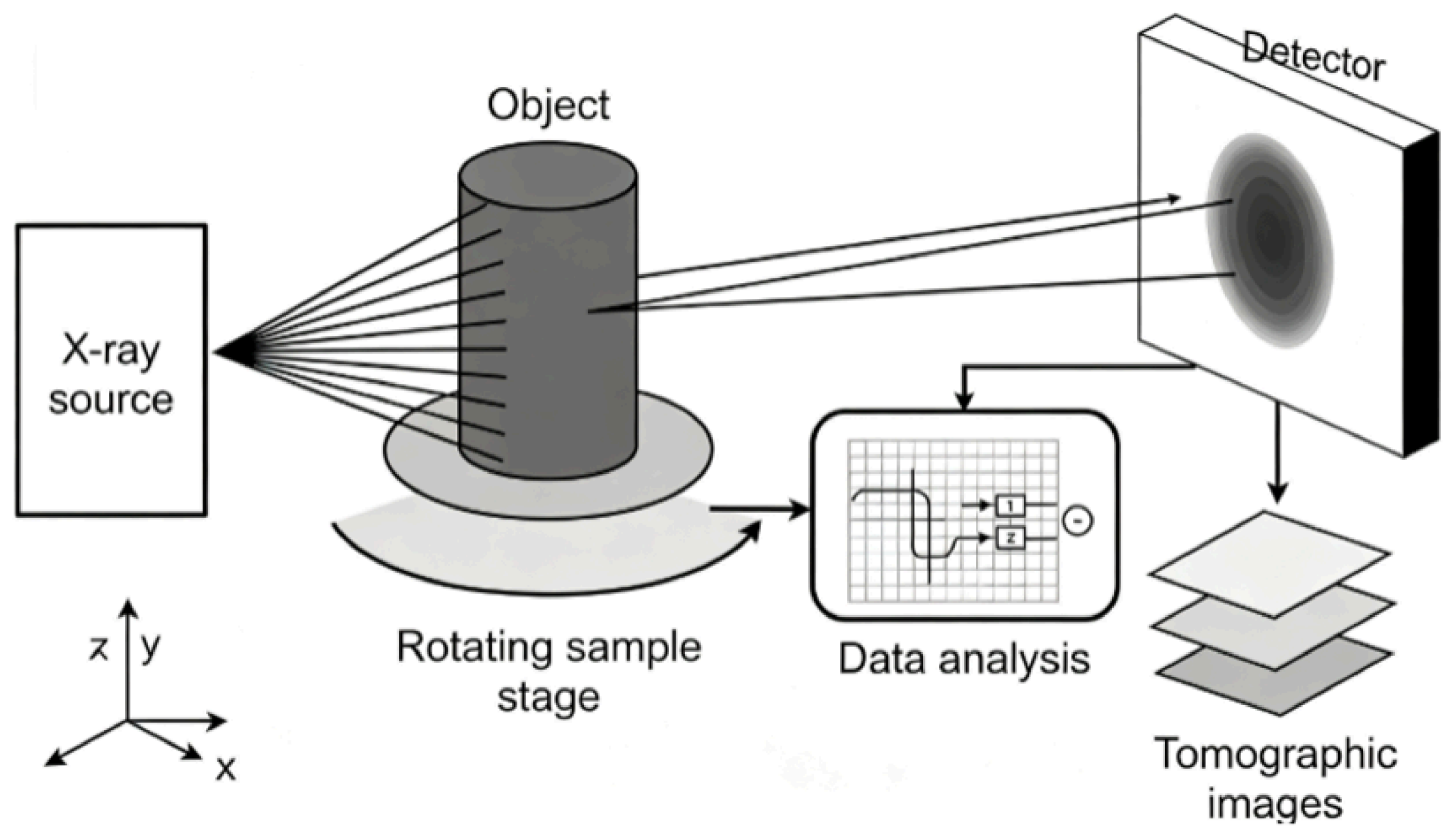
Schematic diagram of the XCT system.

XCT is currently the gold standard technique for the non-destructive quantitative characterization of internal defects in SLM components [57,58]. Its core principle is based on the difference in linear attenuation coefficients of different materials when X-rays penetrate the component. Through multi-angle projection acquisition and three-dimensional reconstruction algorithms, the internal three-dimensional structure of the component can be reconstructed, enabling non-destructive visualization, precise localization, and quantitative statistics of defects such as porosity, cracks, and inclusions [59,60,61]. Compared with ultrasonic testing, XCT features high spatial resolution, direct acquisition of the three-dimensional morphology and spatial distribution of defects, no requirement for couplants, and no restriction by complex internal cavity structures. It serves as the core benchmark technique for verifying the accuracy of other NDT methods, and its working principle is illustrated in Figure 6.

Micro-CT has been extensively applied in the qualitative and quantitative characterization of SLM defects in existing studies. Slotwinski et al. [62] inspected SLM-fabricated Co-Cr alloy disk specimens using XCT and confirmed that the technique can accurately determine the morphology and distribution characteristics of defects. They found that most internal voids are non-spherical and microcrack defects were also observed. However, this study was only conducted on flat specimens of a single cobalt–chromium alloy, without involving complex structures and multi-material systems, resulting in limited generalizability of the conclusions. Kim et al. [63] further performed quantitative analysis on XCT images of SLM cobalt–chromium alloy components and measured the sizes of internal pores and cracks as all being less than 100 μm. Nevertheless, the study failed to establish the correlation between the three-dimensional distribution of defects and mechanical properties, so its guiding value for engineering applications needs to be further strengthened. In practical industrial application scenarios, the detection reliability of XCT is not constant. Its identification accuracy is jointly affected by defect morphology, spatial position, and the structural complexity of components. Industrial CT experiments conducted by Cong [64] on complex SLM components for aerospace applications show that the detection rate of through-hole defects is significantly higher than that of blind holes. Moreover, defects near the center of the component are susceptible to interference from ring artifacts, leading to a notable decline in identification accuracy. This result essentially reflects the inherent technical limitations of industrial CT: the angle between the defect and the beam direction, as well as the presence or absence of an open interface, directly affects the recognizability of attenuation signals. Meanwhile, beam hardening and artifact issues in the central region of the component will also cause fluctuations in the quantitative results of internal defects in complex-structured components. Furthermore, the experiments only covered artificially designed regular pores and did not involve typical irregular SLM defects such as cracks and lack of fusion. This also indicates that the current performance validation of industrial CT is still relatively idealized, and its full-defect-detection capability for real service components still needs to be improved.

Conventional laboratory-scale XCT is limited by its source brightness and imaging mechanism. It generally suffers from low signal-to-noise ratio (SNR) and severe artifact interference during high-resolution inspection, making it difficult to accurately capture the dynamic evolution of defects with varying process parameters. In comparison, synchrotron radiation XCT leverages high-brightness, high-coherence synchrotron radiation sources and exhibits remarkable advantages in imaging contrast, artifact suppression, spatial resolution, and SNR. It enables finer defect morphology characterization and dynamic quantitative analysis of process-defect evolution [65]. Taking advantage of this technical strength, Choo et al. [66] systematically investigated the effects of laser power on the defect characteristics and microstructure of SLM-fabricated 316L stainless steel. They accurately measured that the internal pore volume of specimens increased significantly as the laser power decreased from 380 W to 200 W, clearly revealing the quantitative correlation between energy input and defect evolution. This finding also corroborates the core supporting value of synchrotron radiation XCT in research on defect formation mechanisms. Nevertheless, synchrotron radiation XCT depends on large-scale scientific facility platforms, with prohibitive equipment costs and stringent requirements for inspection environments and operating conditions. Currently, it is only applicable to fundamental mechanism research in laboratories and remains difficult to deploy on a large scale or perform batch component inspection in industrial fields.

Overall, XCT is the core approach for high-precision research on SLM defects and can provide benchmark data for process optimization, failure analysis, and calibration of other testing techniques.

### 3.3. Deep Learning-Based Intelligent Defect-Detection Technology

Defect identification and quantitative analysis in conventional NDT of SLM components largely depend on hand-crafted feature operators and empirical judgment by inspectors [67,68]. This traditional approach is generally constrained by several drawbacks: low data processing efficiency, high subjectivity in interpretation results, and pronounced fluctuations in detection accuracy under complex operating conditions. In recent years, deep learning technology, with its unique strength in automatic feature learning, has been progressively integrated with a variety of testing techniques, including ultrasonic testing, XCT, and optical visual inspection. It can fulfill multiple core tasks, such as defect classification, localization, segmentation, and quantitative statistics, serving as important support for the automation and intelligent upgrading of NDT technologies.

In ultrasonic testing scenarios, deep learning is primarily applied to address the challenge of defect signal feature extraction under high-noise conditions. Conventional methods such as filtering and wavelet analysis suffer from weak adaptability and poor universality. By contrast, convolutional neural networks (CNNs) and temporal network models can automatically learn the joint time-frequency features of defect echoes. They enable accurate classification and localization of multiple defect types while removing background noise, delivering a significantly better performance than hand-crafted signal processing methods [69]. Xu et al. [70] investigated laser ultrasonic testing of SLM-fabricated components with rough surfaces. They verified that CNNs and temporal network models can automatically extract the joint time-frequency features of defect echoes, effectively suppress surface scattering noise, and improve imaging SNR, while achieving precise classification and localization of various defects. The models consistently outperform hand-crafted signal processing approaches in detection performance. Nevertheless, existing models are mostly trained on datasets of laboratory flat specimens, and their transfer adaptability to real-world complex components remains inadequate.

In XCT inspection scenarios, the efficient and accurate segmentation of massive 3D volumetric data is the core bottleneck restricting detection efficiency. Conventional XCT data processing mostly relies on manual annotation or fixed thresholding methods, which suffer from low efficiency and results highly susceptible to operator experience bias. Although traditional unsupervised clustering algorithms represented by K-means can achieve a certain degree of automatic defect identification, they still depend on hand-crafted features. Snell et al. [71] integrated the K-means clustering algorithm into the XCT inspection workflow and realized the automatic identification and quantification of micron-scale voids in multiple SLM alloy specimens, including Ti6Al4V, Ti-5553, IN718, and Haynes 282. This approach effectively reduced the workload of manual annotation and verified the application potential of intelligent algorithms in multi-material systems. Nevertheless, this method essentially still falls into the category of unsupervised clustering and relies on manually predefined features and initial parameters. It has limited identification accuracy for complex defects with irregular morphologies, such as cracks and lack-of-fusion defects, and exhibits insufficient robustness in high-noise image scenarios, with obvious shortcomings in adaptability and generalization performance.

To further break through the performance limits of traditional intelligent algorithms, deep learning semantic segmentation models represented by U-Net and its derivative networks have become the mainstream technical solution in this field, enabling pixel-level automatic identification of multiple defect types, including pores, cracks, and inclusions [72,73]. Guo et al. [74] proposed a semantic segmentation method integrating generative adversarial network (GAN) denoising and attention residual modules for XCT defect detection of SLM-fabricated IN718 components. Its denoising performance outperforms non-local filtering and the RED-CNN algorithm, and its segmentation capability surpasses traditional fully convolutional network (FCN) and U-Net models, achieving a detection accuracy of over 95% for keyhole defects. Compared with traditional algorithms, deep learning models eliminate the need for hand-crafted features. They can autonomously learn multi-dimensional morphological features of defects through data training, delivering significantly improved identification accuracy for irregular lack-of-fusion defects and slender microcracks. Meanwhile, data processing efficiency can be increased several times, which greatly reduces the labor cost and technical threshold of XCT inspection.

In surface defect-detection scenarios, the integration of deep learning and optical imaging technology has significantly improved the identification efficiency and accuracy of micro-defects during offline quality inspection and has become an important technical means for surface quality assessment of SLM-fabricated components. To address the detection challenge posed by highly reflective metal surfaces, Li et al. [75] combined polarization imaging with the SCK-YOLOv5 network. By using polarized light to suppress interference from surface specular reflection, the scheme achieves higher detection accuracy for surface micropores than conventional vision algorithms. Yang et al. [76] proposed a lightweight SLM-YOLO-Light detection framework, which combines visual-domain contrast enhancement and a data augmentation strategy based on a distribution-domain diffusion model. It reaches a 96.3% recognition accuracy for defects including scratches and balling, while the number of model parameters accounts for only 32% of that of the conventional YOLOv5 model, making it more compatible with deployment on industrial edge devices.

In summary, integrating machine learning algorithms such as deep learning with various NDT technologies has become a key direction for realizing intelligent defect identification and quality assessment of metal additive manufactured components, and continues to drive the development of this field toward higher automation and precision.

## 4. Systematic Applicability Analysis and Technical Bottlenecks of Testing Technologies

### 4.1. Multi-Index Quantitative Comparative Analysis of Detection Accuracy, Efficiency, and Cost

SLM components exhibit defects with diverse sizes and types, and various NDT technologies differ greatly in performance and applicable scope. From three perspectives including detection accuracy, inspection efficiency, and deployment cost, this section further incorporates additional indicators such as penetration depth, defect adaptability, and structural compatibility to compare and sort out the mainstream testing technologies mentioned above. The advantages and limitations of each method are summarized to provide references for technical selection in engineering scenarios. The core performance indicators of various technologies are summarized in Table 2.

In terms of detection accuracy, remarkable performance gaps exist among different technologies, which are essentially determined by the physical limits of their detection principles. XCT-related techniques possess the highest resolution. Laboratory micro-CT achieves micron-level resolution and serves as the widely recognized gold standard for quantitative defect characterization. Optical surface inspection ranks second and enables fine characterization of surface topography at the submicron scale. Ultrasonic inspection exhibits relatively moderate accuracy and is mostly adopted for preliminary screening of defects larger than hundreds of micrometers. It should be noted that ultrasonic testing involves an inherent performance trade-off: increasing the transducer frequency can improve resolution yet aggravates acoustic attenuation inside materials and reduces the effective penetration depth, making it impossible to simultaneously achieve high precision and a large penetration depth.

Generally, detection efficiency varies inversely with accuracy [77]. Conventional piezoelectric ultrasonic testing and machine vision deliver the highest inspection efficiency. They support continuous rapid scanning over large component areas and are suitable for batch quality inspection in industrial scenarios. Industrial CT and laser ultrasonic testing achieve moderate efficiency, capable of meeting sampling inspection requirements for small- and medium-batch components. Micro-CT and optical 3D topography inspection have the lowest efficiency. Under high-resolution imaging modes, they can only cover an extremely small field of view. Full-volume inspection of large-sized components often takes several hours or even days, so these methods are only applicable to refined analysis of small laboratory specimens. This discrepancy originates from two aspects. On the one hand, the inherent imaging mechanisms require CT to acquire projection data from multiple angles and conduct three-dimensional reconstruction, while optical 3D topography relies on point-by-point scanning. On the other hand, high-precision inspection generates massive datasets, and subsequent data processing further extends the overall inspection cycle.

In terms of deployment and operation and maintenance costs, the investment gap between different technical solutions can reach one to two orders of magnitude [78,79]. Laboratory micro-CT and synchrotron radiation XCT incur the highest costs, accompanied by stringent requirements for radiation shielding, environmental stability, and professional operators. Laser ultrasonic systems come second in cost, as high-end pulsed lasers and precision optical detection modules usually carry high price tags. Conventional piezoelectric ultrasonic testing, industrial CT, and optical 3D profilometers fall within the medium-cost range. Machine vision requires the lowest investment, featuring simple system construction and low operation barriers, which facilitates large-scale deployment on production lines. The introduction of deep learning algorithms can improve defect recognition accuracy and automation without significantly raising hardware costs and reduce manual interpretation workload. Hence, it acts as an effective approach to enhance the comprehensive detection performance of medium- and low-cost testing schemes.

Overall, SLM NDT technologies are generally restricted by a trade-off triangle among accuracy, efficiency, and cost. High-precision techniques are usually accompanied by low efficiency and high costs, whereas high-efficiency and low-cost methods suffer from evident limitations in detection accuracy and penetration depth. No universal testing technology can simultaneously realize full-scale defect coverage, compatibility with complex structures, high precision, and low cost. In engineering practice, differentiated selection and combined deployment of testing approaches should be carried out according to specific inspection requirements, component structural features, and production batch sizes.

### 4.2. Classification of Applicable Scenarios for Testing Technologies Targeting Different Defect Types

SLM components exhibit a wide variety of defects with pronounced differences in spatial distribution, morphological features, and size ranges. No single detection technology can achieve efficient coverage of all defect types, so corresponding technical solutions must be matched according to the inherent characteristics of defects and specific inspection requirements [80]. Based on the performance boundaries of the aforementioned detection technologies, optimal applicable scenarios can be categorized for four major defect systems, pore-type defects, crack and delamination defects, inclusion and microstructure defects, and surface-forming defects, so as to provide a clear basis for technology selection in engineering applications.

Pores are the most widely distributed defect type in SLM components, with a size range spanning from several micrometers to several millimeters and covering the entire region from the surface to the interior of the component. Detection schemes need to be selected hierarchically according to defect size and burial depth [81,82]. For macroscopic pores (including large keyhole pores) with a size above 0.5 mm and large burial depth, conventional piezoelectric ultrasonic testing is the preferred solution for batch preliminary screening. Such defects show a significant difference in acoustic impedance from the matrix and present distinct echo features; ultrasonic testing can rapidly perform full-area scanning of large-volume components and efficiently locate harmful large pores. However, limited by resolution, it is difficult to accurately distinguish the morphological types of gas pores and lack-of-fusion pores, so this method is only applicable to defect screening and rough localization. For internal micropores at the hundred-micron scale and below, micro-CT is the gold standard for quantitative characterization [83]. With its micron-level spatial resolution, micro-CT can clearly distinguish the morphological differences between spherical gas pores and irregular lack-of-fusion pores, and accurately quantify porosity, size distribution, and spatial orientation, making it a core method for conducting process-defect correlation research and defect hazard assessment [84]. Nevertheless, due to low detection efficiency and high cost, it is only suitable for sampling inspection and refined laboratory analysis. For micropores near the subsurface, laser ultrasound can serve as a supplementary solution: with its high resolution, laser ultrasound breaks through the near-surface blind zone and enables non-contact detection of hundred-micron-scale subsurface pores [36].

Cracks and delaminations are planar structural defects with prominent directional characteristics, and their detection is more challenging than that of pore-type defects. Their technical adaptability is greatly influenced by defect orientation, burial depth, and size [58]. For interlayer delamination defects parallel to the forming plane, conventional piezoelectric ultrasonic testing has the best adaptability. The interface of delamination defects is perpendicular to the incident direction of ultrasound, resulting in echo signals with clear features and high recognizability. This method can efficiently realize rapid screening of delamination defects in large-area components and delivers good accuracy in the quantitative evaluation of delamination area. For internal macroscopic cracks, ultrasonic testing also has preliminary screening capability, but the detection rate is significantly affected by crack orientation: cracks perpendicular to the sound-wave propagation direction produce strong echoes and are easy to identify, while slender cracks parallel to the sound-wave direction are prone to missed detection [85]. For internal micron-scale microcracks, synchrotron radiation CT and high-resolution micro-CT offer higher detection accuracy and can clearly present the three-dimensional morphology and propagation path of cracks [86]; however, conventional micro-CT is limited by slice thickness, and there is still a certain risk of missed detection for flat intergranular microcracks. For surface and near-subsurface microcracks, optical three-dimensional topography testing is a more suitable solution. Optical three-dimensional topography technologies such as laser confocal microscopy can achieve accurate dimensional measurement and morphological characterization of surface microcracks.

Inclusion defects differ from the matrix in density and composition, and their detection adaptability mainly depends on the degree of difference in physical properties between the defects and the matrix [87]. For non-metallic inclusions such as oxides and nitrides, XCT testing is the preferred solution. There is a significant difference in linear attenuation coefficient between the inclusion phase and the metal matrix, resulting in a clear contrast in CT images, which enables intuitive identification of the size, morphology, and spatial distribution of inclusions [88]. Among them, micro-CT can capture submicron ultra-fine inclusions and is the core technology for analyzing the formation mechanism of inclusions. Ultrasonic testing has relatively limited ability to identify inclusions; it can only detect inclusions when they are large in size and have a significant acoustic impedance difference from the matrix, and it is difficult to accurately distinguish inclusions from pores. Therefore, it is generally not used as a special detection method for inclusion defects [89]. For microstructural anomalies such as coarse grains and precipitate segregation, there is currently a lack of direct and efficient NDT methods. Conventional ultrasonic testing can indirectly characterize grain size and microstructure uniformity through changes in sound velocity and acoustic attenuation coefficient, but it can only achieve semi-quantitative evaluation and cannot accurately identify the specific morphology and distribution of microstructures.

Surface-forming defects are all distributed on the outer surface of components, with good detection accessibility. Optical detection technologies are the mainstream adaptive solutions for this type of defect [90]. For macroscopic surface defects such as balling, spatter, scratches, and surface pits, machine vision testing has the best adaptability. With low deployment cost and high detection efficiency, this technology can realize rapid imaging and automatic defect classification on large-area surfaces, fully meeting the requirements of industrial batch offline quality inspection [91]; however, limited by resolution, it is difficult to identify tiny surface defects below ten micrometers. For refined surface quality characterization such as surface roughness, micropores, and microcracks, optical three-dimensional topography technologies such as laser confocal scanning microscopy and white-light interferometers are the first choice. Their submicron-level vertical resolution enables high-precision three-dimensional reconstruction of surface topography and quantitative acquisition of multi-dimensional parameters such as roughness and defect volume, making them core tools for laboratory research on surface quality and process parameter optimization. Nevertheless, their limited detection field of view makes them unsuitable for full-surface inspection of large-size components [92].

Overall, the technical adaptation of defect detection in SLM adheres to a hierarchical strategy of “surface-to-interior progression coupled with coarse-to-fine characterization”: optical techniques are prioritized for surface defect inspection, ultrasonic testing is employed for batch screening of internal macroscopic defects, and XCT is reserved for precise analysis of internal micro-defects and high-accuracy quantitative evaluation. In practical engineering applications, a “pre-screening and refined inspection” protocol is typically implemented, wherein low-cost, high-efficiency methods are first deployed for full-area scanning, followed by high-precision techniques to re-examine and quantify suspicious regions, thereby achieving an optimal trade-off among detection reliability, cost efficiency, and throughput.

## 5. Conclusions and Perspectives

### 5.1. Summary of Research

This review addressed offline NDT of metallic SLM parts, with in situ monitoring considered only to clarify terminology. Its contribution is a defect-to-technology mapping and an accuracy–efficiency–cost selection framework, rather than a new single algorithm or specimen experiment.

Based on the evidence summarized in the preceding sections, the main conclusions are as follows. First, the morphological features and formation mechanisms of SLM defects are directly governed by laser energy input, melt-pool dynamics and interlayer bonding conditions; consequently, defect categories differ fundamentally in size, morphology, spatial distribution, and harm severity, and no single offline NDT technique can achieve full-scale, full-type defect coverage. Second, a clear trade-off exists among detection accuracy, inspection efficiency, and deployment cost: conventional ultrasonic testing and machine vision are efficient and low cost but limited in resolution; laboratory micro-CT provides micron-level reference characterization but suffers from low throughput and high cost; and laser ultrasonics and industrial CT occupy intermediate positions with complementary strengths. Third, deep learning has demonstrably improved defect recognition, segmentation accuracy, and data-processing efficiency across ultrasonic, XCT, and optical inspection scenarios, but its industrial deployment is still constrained by the scarcity of high-quality annotated three-dimensional defect data and by weak cross-material, cross-machine generalization. In addition, simulation and predictive tools—including melt-pool hydrodynamic modeling, thermomechanical analysis, and process-window evaluation—are valuable for explaining defect formation mechanisms and narrowing parameter spaces, but they remain complementary to, rather than substitutes for, experimentally validated offline NDT.

Accordingly, this review supports a hierarchical detection strategy of “surface-to-interior progression coupled with coarse-to-fine characterization”, optical techniques for surface defects, ultrasonic testing for batch screening of internal macro-defects, and XCT for refined quantification of internal micro-defects, implemented through full-area pre-screening followed by localized refined inspection of suspicious regions.

### 5.2. Future Research Directions and Industrialization Trends

Future work in this field is suggested to focus on the following aspects.

(1) Multi-modal detection fusion and hierarchical quality control system construction.

Specifically, the respective advantages of ultrasonic testing, XCT, optical vision, and eddy current techniques can be leveraged to establish a three-tier detection workflow of “surface vision rapid screening—ultrasonic full-area preliminary inspection—XCT localized refined inspection”. Different detection schemes can be matched according to the service classification of SLM components, thereby ensuring reliable inspection results while controlling the overall cost of industrial quality inspection. Concurrently, research on multi-source detection data fusion methods can be advanced, integrating acoustic, radiographic, and optical defect information from both feature-level and decision-level perspectives to enhance the discrimination capability for morphologically similar defects and the recognition accuracy for complex defects, mitigating the issues of defect confusion and elevated miss rates under single-modality conditions. Furthermore, to address the full-process quality control requirements of SLM mass production lines, online optical monitoring during the forming process can be combined with offline NDT sampling inspection after forming, establishing a full-chain quality traceability system interconnecting process, defect, and performance. This would enable closed-loop control encompassing forming anomaly early warning and post-forming defect precise quantification.

(2) Intelligent detection algorithm deepening and optimization for industrial deployment.

Deep learning-based detection models generally suffer from strong dependence on annotated data, insufficient interpretability, and weak cross-scenario generalization capability. To achieve genuine industrial deployment, targeted algorithm optimization constrained by practical application requirements is essential. On one hand, emphasis should be placed on developing few-shot learning, transfer learning, and domain adaptation techniques, enabling model scenario adaptation with limited annotated data. This would alleviate the problems of high annotation costs for SLM three-dimensional defects and insufficient publicly available high-quality datasets, promoting the expansion of intelligent detection models from single-material, single-process scenarios toward multi-material, multi-process scenarios. On the other hand, the interpretability of deep learning models for defect detection needs to be enhanced, utilizing feature visualization, causal reasoning, and related methods to clarify the model’s decision basis and confidence levels, satisfying industrial quality inspection requirements for traceable results and high reliability. At the application deployment level, lightweight model transformation and edge deployment optimization can be advanced, transplanting high-precision detection algorithms onto industrial embedded devices to realize real-time detection data processing and on-site defect judgment, thereby reducing the deployment threshold and computational cost of intelligent detection solutions.

(3) Inspection of complex structures and extreme conditions, coupled with simulation.

Adapted non-contact detection schemes—such as miniaturized ultrasonic phased-array probes, cone-beam CT localized high-resolution imaging, and internal optical endoscopic inspection—should be developed for poorly accessible structures including internal cavities and lattices. For sub-micron ultra-micro defects and grain-boundary microcracks, the application of synchrotron radiation XCT and laser ultrasonic microscopy should be deepened. Critically, offline NDT results should be fed back to simulation and predictive models—melt-pool dynamics, thermomechanical, and process-window models—to reduce their uncertainty and to establish a closed loop among process design, defect prediction, and experimental verification, thereby providing more precise characterization means for revealing the correlation between defect evolution and material properties.

## Figures and Tables

**Table 1:** Typical defects and corresponding formation mechanisms in SLM components.

Defect Type	Core Morphological Characteristics	Main Formation Mechanisms	References
Gas porosity	Spherical or nearly spherical, with pore diameters mostly ranging from several to tens of micrometers; smooth inner walls, regular morphology, and random spatial distribution	Gas trapped in the melt pool cannot escape in time. Gas sources include adsorbed moisture and residual gas on powder particles, entrained shielding gas, and gas generated by oxidation and vaporization of alloying elements. During rapid solidification, the gas is sealed inside the matrix to form closed spherical pores.	[19,20,21]
Lack-of-fusion porosity	Mostly irregular, elongated or crack-like shape with rough edges and sharp angles; wide size range, mainly distributed at interlayer bonding interfaces and overlap zones of adjacent melt tracks	Insufficient laser energy input or low melt track overlap rate leads to incomplete powder melting and ineffective fusion between adjacent melt pools and building layers. Uneven powder spreading and powder agglomeration further aggravate defect generation.	[22,23,24,25]
Keyhole porosity	Mostly large-sized irregular deep holes or through-holes with complex morphology and large pore size variation; often accompanied by local collapse and uneven inner wall features	Metal vaporization under high power density forms a deep keyhole structure. When the keyhole becomes unstable and collapses, the melt cannot backfill in time, forming large, closed pores inside the component.	[26,27,28,29]
Microcracks	Divided into solidification cracks and liquation cracks, distributed along grain boundaries or interdendritic regions; lengths mostly range from micrometers to hundreds of micrometers, with high concealment and propagation tendency	Solidification cracks: grain boundary liquid films are torn under the coupling of solidification shrinkage stress and thermal stress at the final solidification stage. Liquation cracks: low-melting-point phases at grain boundaries in the heat-affected zone remelt, and cracks initiate along grain boundaries during cooling.	[30,31,32,33]
Interlayer delamination	Distributed parallel to the building substrate, presenting sheet-like or band-like gap features; defect width is significantly larger than that of conventional microcracks	Insufficient penetration depth of the upper melt pool prevents effective metallurgical bonding between layers; sustained residual tensile stress aggravates interface debonding; surface morphology defects on previous layers hinder melt spreading and fusion.	[19,20,21]
Non-metallic inclusions	Dominated by hard phases such as oxides, nitrides, and carbides; mostly punctate, flocculent, or irregular block shapes, with sizes ranging from submicron to tens of micrometers, dispersedly distributed in the matrix	Oxide films on powder surfaces enter the melt pool during melting; insufficient shielding atmosphere purity causes metallurgical reactions between high-temperature melt and residual oxygen/nitrogen, generating inclusions in situ.	[34,35,36]
Microstructural abnormality	Characterized by coarse grains, disordered orientation, precipitated phase segregation, and inhomogeneous grain boundary structure; prone to form coarse columnar grains and composition segregation	Extremely high temperature gradient drives grains to grow epitaxially and preferentially along the heat conduction direction; insufficient diffusion of alloying elements during rapid solidification and selective vaporization of low-boiling-point elements lead to uneven composition distribution.	[22,23,24]
Balling and spatter defects	Spherical metal particles and spatter particles adhere to the surface, accompanied by local surface unevenness	Imbalanced laser energy density and melt-pool fluid instability cause melt agglomeration and shrinkage, forming balling defects; metal vapor recoil pressure and shielding gas flow disturbance trigger melt spatter, which adheres to the forming surface.	[25,26,36]
Surface morphology defects	Include surface powder adhesion, melt track undulation, surface pits, scratches, and burrs; overall manifested as increased surface roughness	Caused by the combined effects of uneven powder spreading, unreasonable scanning path, and interlayer fusion differences; the staircase effect and powder sticking effect aggravate surface morphology deterioration with the change in surface inclination angle.	[25,26,35]

**Table 2:** Core performance metrics of various technologies.

Technology Category	Minimum Detectable Defect Size	Typical Inspection Efficiency	Equipment Cost Level	Effective Detection Depth	Main Adaptable Defects	Structural Adaptability
Conventional Piezoelectric Ultrasonic Testing	0.3–0.5 mm	100 cm^2^/min	Medium	20–50 mm	Large-sized pores, lack-of-fusion defects, macro-cracks	Suitable for regular surfaces; limited when dealing with complex curved surfaces and internal cavities [52,53]
Laser Ultrasonic Testing	0.1–0.3 mm	10 cm^2^/min	High	5–20 mm	Micropores, microcracks, subsurface defects	Non-contact testing, compatible with complex curved surfaces, no couplant requirement [54,55]
Laboratory Micro-CT	1–10 μm	0.1 cm^3^/h (high resolution)	Extremely high	Restricted by specimen size, generally <50 mm	All types of internal defects, micron-scale inclusions	Compatible with internal cavities and lattice structures; full 3D coverage for structures [60,62]
Industrial CT	0.1–0.5 mm	10 cm^3^/h	High	Up to several hundred millimeters	Internal defects in medium and large components	Universal structural adaptability, applicable to inspection of large-scale industrial components [64,65]
Optical 3D Topography Inspection	0.1–1 μm	1 cm^2^/min	Medium-high	Surface/subsurface only (<0.1 mm)	Surface micro-defects, surface roughness	Limited field of view; multi-view stitching required for complex curved surfaces [26]
Machine Vision Inspection	5–20 μm	100 cm^2^/min	Low	Surface only	Surface balling, spatter, scratches, pits	Works well for flat and gently curved surfaces; accuracy degraded on highly reflective surfaces [75,76]

## Data Availability

No new data were created or analyzed in this study. Data sharing is not applicable to this article.
